# Aldosterone Effect on Cardiac Structure and Function

**DOI:** 10.2174/011573403X281390240219063817

**Published:** 2024-02-29

**Authors:** Ekhlas Mahmoud Al-Hashedi, Fuad A. Abdu

**Affiliations:** 1 Department of Internal Medicine, Faculty of Medicine and Health Sciences, Sana'a University, Sana'a, Yemen;; 2 Department of Cardiology, First Affiliated Hospital of Xi’an Jiaotong University, Xi’an, Shaanxi, China;; 3 Department of Cardiology, Shanghai Tenth People's Hospital, Tongji University School of Medicine, Shanghai, China

**Keywords:** Aldosterone, cardiac remodelling, primary aldosteronism, essential hypertension, heart failure, atrial fibrillation

## Abstract

**Background:**

Cardiac remodelling could be a key mechanism in aldosterone-mediated cardiovascular morbidity and mortality. Experimental and clinical evidence has demonstrated that aldosterone causes cardiac structural remodelling and dysfunction by its pro-fibrotic and pro-hypertrophic effects, which result mainly from the direct effects on myocardial collagen deposition, inflammation, and oxidative stress. Clinical studies have investigated the aldosterone effects on the heart in different clinical conditions, including general population, essential hypertension, primary aldosteronism, heart failure, and atrial fibrillation. Robust findings indicate that aldosterone or the activation of the cardiac mineralocorticoid receptor can cause damage to myocardial tissue by mechanisms independent of the blood pressure, leading to tissue hypertrophy, fibrosis, and dysfunction.

**Conclusion:**

Aldosterone-mediated cardiovascular morbidity and mortality mainly result from cardiac structural and functional alterations. In different clinical settings, aldosterone can induce cardiac structural remodelling and dysfunction *via* several pathological mechanisms, including cardiac fibrosis, inflammation, and oxidative stress. Aldosterone antagonists could effectively decrease or reverse the detrimental aldosterone-mediated changes in the heart.

## 1 INTRODUCTION

The mineralocorticoid aldosterone is one of the effector hormones of the renin-angiotensin-aldosterone system. It is synthesized in the glomerular zone of the adrenal cortex [1]. One of the aldosterone action targets is cardiomyocytes expressing mineralocorticoid receptors (MR) [2]. Aldosterone is also made locally in various tissues in the body, including the myocardium [3]. Locally-made aldosterone may act on local MR to mediate local adverse effects. For this reason, baseline plasma aldosterone values appear to only poorly predict the efficacy of aldosterone blockade [4]. Angiotensin II acts as the primary stimulus for aldosterone secretion [5]. However, aldosterone production may escape despite the angiotensin-converting enzyme (ACE) inhibition, and this could be due to the non-ACE-dependent production of angiotensin II in addition to the ACE-dependent mechanisms [6-8]. Moreover, aldosterone may be synthesized *via* angiotensin II-independent pathways involving adrenocorticotropic hormone and potassium [9]. Therefore, even if ACE inhibitors and angiotensin receptor blockers are largely beneficial for preventing cardiac remodelling [10], these medications would not completely suppress aldosterone-specific effects, and blocking aldosterone receptors preventing aldosterone-mediated effects on the heart appears crucial [11-13].

Aldosterone, together with high dietary sodium intake, stimulates inflammatory reactions, extracellular matrix formation, cellular hypertrophy, and apoptosis in the heart, vessels, and kidneys [14]. There are direct and indirect (*i.e*., hypertension-mediated) mechanisms by which aldosterone leads to cardiac structural remodelling and dysfunction. Aldosterone exhibits pro-fibrotic and pro-hypertrophic actions independent of blood pressure and plasma volume [15]. The observed reversal of myocardial hypertrophy and fibrosis by non-antihypertensive dosages of the MR antagonist spironolactone supports these findings [16]. Inappropriate production of aldosterone hormone, as characterised by primary aldosteronism, is associated with a higher rate of structural and functional cardiac abnormalities [17, 18]. However, according to a growing body of evidence, aldosterone, even within the physiological range, is a significant cardiovascular risk factor [19]. This article reviews updated clinical studies addressing aldosterone's role in affecting cardiac structure and function in the general population, essential hypertension, primary aldosteronism, heart failure, and atrial fibrillation.

## 2 CARDIAC EFFECTS OF ALDOSTERONE IN THE GENERAL POPULATION

Significant associations were identified between changes in left ventricular mass (LVM) and changes in urine aldosterone concentration and between changes in LVM and aldosterone response to angiotensin II in young, healthy Caucasian men [20]. Additionally, in a cohort study of 1575 patients with cardiovascular risk factors and preserved LV ejection fraction, Edelmann *et al.* [21] found that higher plasma aldosterone levels were related to relative wall thickness (RWT) and left ventricular mass index (LVMI) in women and independently associated with concentric left ventricular hypertrophy (LVH) in both genders. Likewise, another large community-based cohort study found an association between aldosterone and the aldosterone-to-renin ratio with both concentric and eccentric LVH [22]. On the other hand, a large cohort of subjects free of Myocardial Infarction and overt heart failure reported an association of serum aldosterone with LV concentric remodelling in women but not men [23].

## 3 CARDIAC EFFECTS OF ALDOSTERONE IN ESSENTIAL HYPERTENSION PATIENTS

Cardiac remodelling in essential hypertension is characterized by myocyte hypertrophy and enhanced interstitial fibrosis due to increased collagen synthesis and unaltered or decreased collagen degradation [24]. Circulating biomarkers of myocardial fibrosis are associated with hypertensive heart disease, and myocardial fibrosis appears to precede the development of LVH [25].

Numerous studies examined the relationship between aldosterone and LV remodelling and geometric patterns in individuals with essential hypertension [26-28]. For instance, one report enrolled 75 consecutive patients with never-treated essential hypertension and 45 normal controls, emphasizing the association between serum aldosterone and LVMI, RWT, and global longitudinal strain. In addition, aldosterone level was related to diastolic function, which is consistent with aldosterone's stimulating effect on myocardial fibrosis [29, 30].

Interestingly, plasma aldosterone and indicators of a prothrombotic state, such as fibrinogen, are significantly correlated in essential hypertension patients [31]. Catena *et al.* [32] evaluated the correlation between plasma aldosterone, haemostatic variables, and cardiac morphology in 205 middle-aged individuals with varying degrees of essential hypertension. They found that elevated LVMI was related to higher plasma aldosterone levels, and this association was partly due to elevated fibrinogen levels.

Furthermore, a study of essential hypertension patients with metabolic syndrome reported that elevated plasma aldosterone concentration related to obesity might assist in explaining the increased LVM noted in association with metabolic syndrome and contribute to increasing the cardiovascular risk associated with metabolic syndrome [33].

## 4 CARDIAC EFFECTS OF ALDOSTERONE IN PRIMARY ALDOSTERONISM

Excess aldosterone is associated with increased cardiovascular risk in primary aldosteronism patients. A meta-analysis of 31 studies, including 3838 primary aldosteronism patients and 9284 essential hypertension patients, revealed that primary aldosteronism patients had a higher risk of cardiovascular complications, such as stroke, coronary artery disease, atrial fibrillation, heart failure, and LVH, compared with patients with essential hypertension [34].

In primary aldosteronism, aldosterone excess is relatively independent of the renin-angiotensin system and only weakly suppressed by sodium loading [35]. As a result, primary aldosteronism provides a unique clinical model for studying the effects of excess aldosterone on the heart because these effects are distinct from those of the renin-angiotensin axis.

In patients with primary aldosteronism, aldosterone induces LV remodelling through cardiac fibrosis and hypertrophy. LV remodelling is influenced by the production of reactive oxygen species, inflammation, pro-fibrotic mediators, collagen synthesis, myosin production, and phosphorylation induced by excessive aldosterone. Poor diastolic and systolic function and worse clinical outcomes are linked to the structural changes following LV remodelling [36].

In echocardiographic studies, primary aldosteronism patients have more frequent and progressive LVH and worse LV diastolic function than matched essential hypertension patients [17, 37, 38]. Similarly, in a cardiac magnetic resonance imaging study, primary aldosteronism patients exhibited a greater degree of ventricular hypertrophy and enlargement, as well as myocardial fibrosis, than those with essential hypertension [18]. Moreover, patients with primary aldosteronism presented a significantly lower longitudinal systolic strain in addition to a higher LVMI and RWT compared to essential hypertension patients [39].

Interestingly, aldosterone excess induces deleterious cardiac effects in the presence of high dietary salt intake [15], which plays a vital role in cardiac remodelling even after successful treatment for primary aldosteronism [40]. A case-control study enrolled primary aldosteronism patients and control patients with essential hypertension who were matched for age, gender, duration of hypertension, and 24-hour systolic and diastolic blood pressure, found that 24-hour urinary sodium excretion was an independent predictor for LV wall thickness and LVM among the patients with primary aldosteronism but not in patients with essential hypertension. Consequently, dietary salt is crucial in determining the severity of cardiac injury in patients with primary aldosteronism. Therefore, dietary salt restriction might reduce cardiovascular risk [41].

Aldosterone excess is associated with cardiac structural and functional changes even without hypertension. This claim is proved by the finding of a study in normotensive individuals with genetically proven familial hyperaldosteronism type I, in which aldosterone excess was related to higher LV wall thicknesses and reduced diastolic function, as reflected by lower mitral early peak velocities, ratios of early to late peak diastolic transmitral flow velocity, and myocardial early peak velocities [42].

Aldosterone-induced TIMP-1 expression, a key factor of myocardial fibrosis, resulted in enhanced collagen accumulation *via* the suppression of matrix metalloproteinase-1 activity [43]. A clinical study prospectively investigated 27 patients with aldosterone-producing adenoma preparing for adrenalectomy and 27 patients with essential hypertension. Patients in the aldosterone-producing adenoma group were also followed one year after receiving an adrenalectomy. The study found that TIMP-1 was associated with aldosterone-induced diastolic dysfunction caused by excess aldosterone [44].

Early detection and targeted treatment of primary aldosteronism normalize blood pressure and reverse detrimental LV changes in the long term [45]. A report prospectively analyzed patients with aldosterone-producing adenoma who underwent adrenalectomy showed that LVMI in patients with primary aldosteronism declined significantly after adrenalectomy [46]. Similarly, a meta-analysis of 14 studies, including 629 hypertensive individuals with primary aldosteronism, concluded that adrenalectomy exerts a beneficial effect on LV structure and geometry by lowering the burden of LVH and LV concentric geometry [47].

In addition, a 7-year [48] and a 3-year [49] follow-up study exhibited that both adrenalectomy and spironolactone therapy for primary aldosteronism patients results in a significant and comparable reduction in LVM. Notably, both reports have demonstrated that the decrease in LVM in primary aldosteronism patients appears earlier following adrenalectomy than spironolactone treatment. Although cardiac structural alterations, notably LVH, can be reversed after surgical or medical therapy for primary aldosteronism [45], the effects of primary aldosteronism treatments on diastolic dysfunction are still inconsistent [48].

## 5 CARDIAC EFFECTS OF ALDOSTERONE IN HEART FAILURE PATIENTS

Patients with heart failure have elevated levels of aldosterone that can exceed twenty times the normal limits [50]. Increases in aldosterone levels are linked to higher heart failure mortality [51]. Regardless of ejection fraction, aldosterone upregulation is a well-documented pathophysiologic phenomenon that promotes myocardial fibrosis, LVH, endothelial dysfunction, and inappropriate salt handling in patients with heart failure [52].

### 5.1 Aldosterone and Heart Failure with Reduced Ejection Fraction

Heart failure with Reduced Ejection Fraction (HFrEF) pathogenesis involves several mechanisms, including d+ysregulated neurohumoral stimulation, high hemodynamic overload, ventricular remodelling, and extracellular matrix abnormalities [53].

Local collagen deposition causes cardiac fibrosis, which reduces myocardial contractility. As a result, biomarkers of myocardial fibrosis are linked to LV systolic and diastolic dysfunction in HFrEF, and abnormal collagen type I metabolism with excessive degradation has independent prognostic significance for all-cause and cardiovascular mortality in these patients [54].

Table **1** summarizes the clinical trial studies of the effect of MR antagonists in heart failure patients. RALES trial (Randomized Aldactone Evaluation Study) was the first to show that adding spironolactone to conventional medication decreased mortality and morbidity in patients with advanced HFrEF (New York Heart Association (NYHA) class III-IV) [13]. These results were further extended by the EPHESUS trial (Eplerenone Post-Acute Myocardial Infarction Heart Failure Efficacy and Survival Study) in heart failure patients after myocardial infarction [55] and the EMPHASIS-HF trial (Eplerenone in Mild Patients Hospitalization And Survival Study in Heart Failure) in patients with NYHA class II HFrEF [56], both trials showed that the MR antagonist eplerenone, in addition to standard therapy, caused a highly substantial decrease in mortality and heart failure hospitalizations in those individuals. Additionally, finerenone, a non-steroidal selective MR antagonist, showed equivalent benefits to eplerenone in HFrEF patients with diabetes mellitus and/or chronic renal disease who required hospitalization [57]. As a result, MR antagonists are a fundamental treatment in those with HFrEF and are class I recommendations in current HF treatment guidelines [58, 59].

### 5.2 Aldosterone and Heart Failure with Preserved Ejection Fraction

Heart failure with preserved ejection fraction (HFpEF) is characterized by abnormal hemodynamics and pathophysiological alterations in cardiac structure and function [60]. It is mainly accompanied by LV diastolic dysfunction.

Myocardial fibrosis, a key component of HFpEF pathophysiology, results from increased collagen synthesis (or decreased collagen breakdown), inflammation, and oxidative stress [61]. The increase in myocardial collagen content contributes significantly to the development of LV diastolic dysfunction by influencing both LV relaxation and stiffness [62]. Aldosterone and its MRs are potentially implicated in this setting. Elevated aldosterone is related to an increased risk of heart failure rehospitalization and all-cause death in individuals with HFpEF [63].

MR antagonists are of particular interest in the treatment of HFpEF because of their effects on interstitial fibrosis, extracellular matrix expansion, myocardial stiffness, and vascular function. Randomized controlled trials (RCTs) have shown that MR antagonist administration in HFpEF helps to improve tissue relaxation and favorable changes in collagen turnover markers [64, 65]. Another report revealed that spironolactone treatment lowers myocardial fibrosis *via* decreasing serum collagen type I marker in heart failure with mid-range ejection fraction (HFmrEF) and HFpEF. It also reduced collagen type III marker levels but only in patients with HFpEF [66]. These reports indicate that MR antagonists have disease-modifying potential in HFpEF patients.

A meta-analysis of 14 RCTs, including 6428 patients with HFpEF or MI with preserved ejection fraction, found that MR antagonist therapy led to a reduction in heart failure hospitalizations by 17%, improved diastolic function, reversed cardiac remodelling, and enhanced quality of life, but did not reduce all-cause mortality [67]. However, TOPCAT trial (Treatment of Preserved Cardiac Function Heart Failure with an Aldosterone Antagonist) in HFpEF [68, 69] and ALBATROSS trial (Aldosterone Lethal effects Blocked in Acute Myocardial Infarction Treated with or without Reperfusion to improve Outcome and Survival at Six months follow-up) in patients with acute myocardial infarction, 92% of whom presented without heart failure [70], both trials failed to support these findings about the effectiveness of MR antagonist in reducing cardiovascular mortality and heart failure hospitalizations (Table **1**). These discrepancies could be due to the heterogeneous population of ALBATROSS in terms of cardiac function, which limits any analysis or comparison to other studies [71] and heterogeneity of practice patterns and/or accuracy of diagnostic procedures to diagnose patients with relatively preserved ejection fraction in TOPCAT, which preclude reaching reliable results [72].

## 6 ALDOSTERONE AND ATRIAL FIBRILLATION

A mounting body of evidence indicates that atrial structural and electrical remodelling creates the basis for atrial fibrillation development. Notably, atrial fibrosis represents the main characteristic of atrial structural and electrical remodelling [73].

Aldosterone levels have been reported to be increased in patients with persistent atrial fibrillation and to fall after sinus rhythm is restored [74]. Atrial MR expression, in addition, is elevated in patients with atrial fibrillation [75]. On the other hand, the chronic increase in the aldosterone concentration in terms of primary aldosteronism contributes to the increased risk of atrial fibrillation [76].

Evidence indicates that aldosterone might trigger atrial arrhythmias characterized by atrial fibrosis, myocyte hypertrophy, conduction abnormalities, and ion channel remodelling [75, 77]. Pro-arrhythmic effects of aldosterone may also be mediated by increased sympathetic drive, slowed myocardial norepinephrine uptake, disrupted baroreceptor function, decreased heart rate variability, and alterations in electrolyte homeostasis [78].

Aldosterone increases cardiac fibrosis and inflammation and causes oxidative stress [79]. It also increases blood pressure and induces cardiomyocyte growth, necrosis, and reparative cardiac collagen deposition. These changes, which cause LVH and fibrosis [80], imply LV stiffening, impaired LV filling, atrial stretching, and left atrium dilatation, *i.e.,* processes favoring re-entry mechanisms [81].

Aldosterone effects on the heart are not limited to hypertrophy and fibrosis, as they extend to the modification of ionic currents and electrophysiological properties of cardiomyocytes [82, 83]. Aldosteronism can produce severe hypokalemia, which prolongs the time of atrioventricular conduction and atrioventricular re-entry mechanisms and enhances the risk of developing atrial fibrillation [81]. Aldosterone also triggers direct electrophysiological changes in cardiomyocytes that promote atrial fibrillation [84]. Additionally, aldosterone shortened the action potential of the left atrium and doubled the time required for atrial fibrillation to convert to sinus rhythm spontaneously [85]. Hence, it is proposed that aldosterone per se established a trigger for atrial arrhythmias independent of changes in LV afterload.

The ALDO-POAF (ALDOsterone for predicting Postoperative Atrial Fibrillation) study found that preoperative aldosterone levels might be a helpful biomarker for identifying patients at a higher risk of developing postoperative atrial fibrillation [86]. Moreover, the results of the PAPPHY (Prospective Appraisal on the Prevalence of Primary Aldosteronism in Hypertensive) study supported the link of primary aldosteronism with atrial fibrillation: Hypertensive patients with atrial fibrillation were found to have undiagnosed primary aldosteronism in 42% of cases, and the condition was surgically treated in half of such cases [87].

Two meta-analyses investigated the impact of MR antagonists on atrial fibrillation occurrence (of any type and context). They found that patients taking MR antagonists had a much lower overall risk of developing atrial fibrillation [88, 89]. Additionally, after radiofrequency catheter ablation in atrial fibrillation patients, spironolactone can reduce atrial fibrillation recurrence and enhance LV function [90].

## CONCLUSION

In addition to experimental evidence, clinical studies in different clinical settings firmly show that aldosterone has a significant and independent role in triggering deleterious cardiac effects. Several mechanisms can result in aldosterone-mediated changes in the heart, including cardiac fibrosis, inflammation, and oxidative stress. Salt intake and other factors might facilitate these alterations. Aldosterone might cause several cardiac structural remodelling and dysfunction, including LVH and diastolic dysfunction. These aldosterone-induced cardiac changes can be independent of their impact on blood pressure. Thus, it is crucial to address aldosterone excess and not just blood pressure in the relevant clinical conditions. MR blockade could effectively decrease or reverse these harmful consequences.

## Figures and Tables

**Table 1:** Randomized controlled trials of the effect of mineralocorticoid receptor antagonists in heart failure patients.

**Trial**	**RALES**		**EPHESUS**	**EMPHASIS-HF**	**ARTS-HF**		**ALDO-DHF**	**RAAM-PEF**	**TOPCAT**		**ALBATROSS**
**Year ^a^**	1999		2003	2011	2016		2013	2011	2014		2016
**Primary endpoints**	All-cause or CV mortality, hospitalization for HF		All-cause or CV mortality, hospitalization for CV events	CV mortality, hospitalization for HF	30% ↓in plasma NT-proBNP from baseline to day 90		Changes in exercise capacity and diastolic function	changes in 6-minute walk distance	CV mortality, aborted cardiac arrest, or hospitalization for HF		Death, resuscitated cardiac arrest, ventricular arrhythmia, indication for implantable defibrillator, new or worsened HF
**No. of patients**	1663		6642	2737	1066		422	44	3445		1603
**Included patients**	HF NYHA III–IV, EF < 35%		Post-MI (within 3–14 days), HF or diabetes, NYHA I–IV, EF < 40%	HF NYHA II, EF < 35%, ˂ 55 years	HF EF≤ 40%, T2DM and/or CKD (eGFR of ˃30 mL/min/1.73 m^2^ in patients with T2DM and 30– 60 mL/min/1.73 m^2^ in patients without T2DM), ≥18 years,		HF NYHA II/III, EF ˃50%, evidence of diastolic dysfunction, ≥50 years	HF NYHA II or III, EF ≥50%, BNP ≥100 pg/mL, ≥18 years	HF NYHA I–IV, EF ≥ 45%), ≥50 years		STEMI or high-risk non-STEMI regardless of HF, ≥18 years,
**Age**	65 ±12 years (Mean± SD)		64 ± 12 years (Mean± SD)	68.7 ± 7.7 years (Mean± SD)	Mean age range from 69.2 to 72.5 years in different groups (SD 9.7–10.6 years)		67 ± 8 years (Mean± SD)	- Eplerenone group, 72.2 ±9.8 years - Placebo group, 68.7±9.1 years (Mean± SD)	68.7 years (Median) in both spironolactone and placebo group		58 years (Median) in both MRA and standard therapy group
**MRA**	Spironolactone		Eplerenone	Eplerenone	Finerenone and eplerenone		Spironolactone	Eplerenone	Spironolactone		Single intravenous bolus of K+ canreonate, then oral spironolactone
**Follow-up duration**	24 months		16 months	21 months	90 days		12–18 months	6 months	3.3 years		6 months
**Main findings of primary endpoints**	↓ all-cause mortality, CV mortality, hospitalization for HF in Spironolactone group	↓all-cause mortality, CV mortality, hospitalization for HF in eplerenone group		↓CV mortality, hospitalization for HF in eplerenone group		Finerenone reduced levels of NT-proBNP similar to eplerenone	Improved LV diastolic function, unchanged maximal exercise capacity	No improvement in exercise capacity	No significant reduction in CV death, aborted cardiac arrest, or hospitalization for HF	No significant reduction in the death from any cause, or any other primary endpoints in MRA group	

**Abbreviations:** CV, cardiovascular; HF, heart failure; NT-proBNP, N-terminal pro-B-type natriuretic peptide; EF, ejection fraction; NYHA, New York Heart Association Functional Classification; MI, myocardial infarction; LV, left ventricle; MRA, mineralocorticoid receptor antagonists.

**Note:** ^a^Year of published results.

## LIST OF ABBREVIATIONS

MR: Mineralocorticoid Receptors
ACE: Angiotensin-Converting Enzyme
RWT: Relative Wall Thickness
RCTs: Randomized Controlled Trials
